# Correlates of attitudes toward wife beating among married/partnered individuals in Lao PDR: results from the 2023 Multiple Indicator Cluster Survey

**DOI:** 10.3389/fpubh.2026.1741590

**Published:** 2026-04-07

**Authors:** Masood Ali Shaikh

**Affiliations:** Department of Medicine, College of Medicine, Korea University, Seoul, Republic of Korea

**Keywords:** wife beating attitudes, MICS, Lao PDR, intimate partner violence, alcohol use

## Abstract

**Background:**

The pervasiveness of wife-beating attitudes (WBA) acceptance is alarming and significantly influences intimate partner violence (IPV). Understanding correlates of this acceptance is important for designing targeted interventions and policies to reduce IPV.

**Methods:**

Using de-identified data from the Lao PDR Multiple Indicator Cluster Survey 2023, a secondary analysis of WBA and their correlates in currently married/partnered men and women aged 15–49 years was conducted, with separate analyses performed for each sex. The correlates analyzed were age, educational attainment, residence, household wealth, number of children, multiple wives/partners, media exposure, alcohol use in the past 30 days, and age difference between a woman and her husband/partner. Simple binary logistic regression models were used to select correlates at a *p* ≤ 0.20 for inclusion in the multivariable models to analyze associations with WBA acceptance; in the multivariable models, a *p* < 0.05 was deemed statistically significant.

**Results:**

The prevalence of WBA acceptance was 13.51% in women and 11.02% in men. For both sexes, the most commonly accepted reason for wife beating was if the wife argues with her husband, and the least common was if she burns the food. In the multivariable models, exposure to media was statistically significantly associated with lower odds of WBA acceptance in both sexes. In men, alcohol use in the past 30 days was associated with increased odds of WBA acceptance, while in women, having a husband/partner 10 years or more older was associated with decreased odds of such acceptance.

**Conclusion:**

The prevalence of WBA acceptance in double digits is concerning in Laos, suggesting the need for targeted health education and promotion campaigns to raise awareness about adverse and harmful attitudes, with a focus on both sexes and women’s rights. Strategies to increase media exposure and use of media as a vehicle for tackling such attitudes hold promise.

## Introduction

Attitudes toward wife beating reflect deeply entrenched gender norms. However, attitudes are necessary for, but not necessarily sufficient enough to result in, behaviors (1, 2). The pervasiveness of intimate partner violence (IPV) reflects global gender inequality and a public health governance failure (3). The United Nations Development Programme’s (UNDP), 2023 Gender Social Norms Index (GNSI), reported that 90% of people hold gender-based biases, and 25% believe that beating your wife can be justified (4). For many developing countries, cross-sectional Demographic and Health Surveys (DHSs) provide the only nationally and sub-nationally representative metrics on the prevalence and correlates of IPV, including attitudes on wife beating. Several studies have shown a positive association between women who report IPV and their accepting attitudes toward wife beating using DHS data (5–7); however, a lack of this association has also been empirically documented (8, 9). DHSs inquire about wife-beating attitudes by asking five questions on its acceptance in terms of justifiability: when a wife neglects children, refuses sex with her husband, argues with her husband, burns food, or goes out without informing her husband. The Multiple Indicator Cluster Surveys (MICS) are conducted by the United Nations Children’s Fund (UNICEF) that also inquire about wife-beating attitudes using the same questions. But, in some countries, such as Tonga and Zimbabwe, additional questions have also been asked about experiencing physical and sexual IPV, including victims’ help-seeking behavior.

Several studies using MICS data have analyzed the correlates of wife-beating attitudes (WBA). A study from the Punjab province of Pakistan reported an association of low WBA acceptance in women with higher educational level, higher household wealth, and more media exposure (10). Using MICS data from Guyana, it was reported that women living in rural areas, hailing from lower wealth quantiles, with low educational attainment and low media exposure were associated with higher acceptance of WBA (11). Additionally, ethnicity and geographic region/location were also significantly associated with WBA acceptance. However, this report used 10 questions pertaining to WBAs, including the standard five. Similarly, low educational attainment, lower household wealth, and rural residency were also found to be associated with acceptance of WBA by women in Bangladesh, using MICS data (12). Another study based on pooled data analysis using MICS data from Nepal, Lao PDR (MICS 2017), and Bangladesh found higher educational attainment to be the most important correlate of WBA acceptance in women, in addition to increased household wealth (13). Higher educational attainment and wealth status were also reported to be associated with low WBA acceptance by women in Eswatini and Vietnam (14, 15). Using MICS data for women and men from 39 and 13 countries, respectively, it was reported that in general lowest wealth quantile, rural residency status, low educational attainment, and younger age were associated with higher WBA acceptance (16).

Similarly, several studies have analyzed the correlates of WBA using DHS data. A study using DHS data from twenty low- and lower-middle-income countries reported a 4.84% cumulative prevalence of wife-beating attitudes acceptance in male adolescents (15–19-year-old) with a wide variation among countries ranging from 16.11% in Timor-Leste to 1.56% in Zimbabwe (17). It reported that residents of rural areas were more likely to report acceptance of WBA while higher educational attainment was associated with lower acceptance rates. Another study based on 21 sub-Saharan African countries reported the association of polygyny with higher acceptance of WBAs in women (18). The association of interparental violence exposure in women with higher WBA acceptance was reported by another study in 22 out of 26 sub-Saharan African countries (19). A study based on two recent DHSs conducted in Pakistan reported association between higher educational attainment, wealth status and urban residency with lower acceptance of WBA, in both women and men aged 15–49 years (20). Regional variations in the country and higher WBA acceptance among women compared with men were also reported. Based on Ghana DHS, WBA was studied among women and men aged 15–25-years-old. Higher wealth and reading newspapers at least once a week were associated with lower odds of WBA acceptance in men. While in women, older age, higher educational attainment, higher wealth, and reading newspapers at least once a week were associated with lower odds of accepting WBA; region of residence was statistically significant for both genders as well (21). The association of increased wealth, higher educational attainment, urban residency, and media access with lower odds of WBA acceptance in both genders was also reported by another study using DHS data from 17 sub-Saharan African countries (22).

A pooled analysis of men aged 15–49 from 10 East African countries found that increasing age, higher educational attainment, and higher wealth quantiles were associated with lower odds of WBA acceptance (23). However, men with more than one wife versus one wife, rural residents, and working men versus not working were more likely to have higher WBA acceptance. Interestingly, there was no statistically significant association with media exposure and number of living children. One study using both MICS and DHS data from 1999 to 2022 for both sexes from 83 countries reported that women were more accepting of WBA, but such attitudes have been declining in both sexes, more so in women (24).

The Lao People’s Democratic Republic (DPR) is a southeast Asian country that has carried out three MICSs, although no DHS. The Lao Social Indicator Survey (LSIS) I, II, and III are Multiple Indicator Cluster Surveys (MICS) implemented under the LSIS name in Lao PDR in 2011–12, 2017, and 2023. The LSIS I, II, and III, respectively, reported 58.2, 29.5, and 12.5% acceptance of WBA in women aged 15–49 years. While in men, WBA acceptance was 49.1, 16.2, and 10.6%, respectively, in LSIS I, II, and III (25).

In Laos, a nationally representative survey of intimate partner violence (IPV) was conducted in 2014 and reported lifetime physical IPV prevalence of 11.6% in ever-partnered women committed by their husbands/partners, with essentially no discernable difference between urban (12.0%) and rural (12.4%) areas (26). However, this survey did not include the five wife beating attitudinal questions that are used in the DHS and MICS.

A study based on the 2011–2012 Lao Social Indicator survey (LSIS) reported that acceptance of WBA in women was associated with decreased probability of receiving antenatal care, postnatal care, and deliveries assisted by trained medical personnel (27). A mixed methods study based on 200 married couples from Savannakhet province reported that 89.8% of women and 86.3% of men accepted wife battering in some or most cases (28).

The LSIS III is the most recent nationally and sub-nationally representative cross-sectional survey on WBA in the Lao PDR. There are no studies on the correlates of WBA among women and men in Lao PDR using a multivariable model with the LSIS III (MICS 2023) dataset.

In this study, LSIS III data was leveraged for secondary analysis to study the WBA attitudes and their correlates in currently married or partnered women and men, separately and to show how different demographic, socioeconomic, and other factors are associated with WBA acceptance in the country. Social norms related to gender roles, family harmony, and household authority may influence WBAs in Lao PDR. Although important for a more nuanced understanding of WBA dynamics, this quantitative analysis focused on WBA correlates available in the MICS rather than examining the cultural mechanisms underpinning such attitudes.

## Methods

### Study area and data source

The Lao Social Indicator Survey III (LSIS III) was conducted in 2023 by the Ministry of Health, the Ministry of Education and Sports, and the Lao Statistics Bureau of the Ministry of Planning and Investment. The UNICEF Global MICS Team, and UNFPA, among others provided technical support. The sample was designed to provide national and sub-nationally representative estimates. The sampling frame was based on the Lao Statistics Bureau’s 2021 village register. A two-stage sampling design was used. In the first stage, villages were systematically selected from provinces with probability proportional to size and, in the second stage, a systematic sample of 20 households were selected. An individual women and men’s questionnaire—based on MICS6 standard questionnaires—was, respectively, administered in each household to all women aged 15–49 and to every second household. Questionnaires were administered using Computer-Assisted Personal Interviewing (CAPI) from March to August 2023. LSIS III, 2023 details on the survey methodology and questionnaires used are available in the freely downloadable country report available on the UNICEF website (29).

The Lao National Ethic Committee for Health Research granted ethical approval. Consent was obtained verbally from each adult respondent, while guardian consent was obtained prior to assent for individuals aged 15–17 years. Access to LSIS III, 2023, deidentified data for secondary analysis was granted to author after free registration on the UNICEF MICS website (30).

### Study variables

The standard MICS6 questionnaires were used for LSIS III. Details on the derivation and use of variables used in this study are described below.

### Outcome variable

LSIS III, 2023 asked respondents the following five specific questions on the justification of a husband hitting or beating his wife to determine WBA:

1. *If she goes out without telling him?*
2. *If she neglects the children?*
3. *If she argues with him?*
4. *If she refuses to have sex with him?*
5. *If she burns the food?*

Answer options included either “yes,” “no,” or “do not know.” A new composite binary variable was created that was coded as “yes” if the respondent answered affirmatively to any one or more of the five WBA questions and no otherwise including when the answer was “do not know.” The MICS Lao PDR used this approach to report WBA prevalence among respondents who are “currently in union,” meaning those who are “currently married or living together with someone as if married” (29).

### Explanatory variables

Several studies have reported on the association of IPV with WBA and other sociodemographic and cultural correlates. In this study, 11 correlates of WBA were examined for women and 10 for men. These included age (7 categories of 5-year intervals); educational attainment (4 categories of none/pre-primary, primary, secondary, and post-secondary/higher); ethnicity of the household head (4 categories of Lao-Tai, Mon-Khymer, Hmong-Mein, and Chinese-Tibetan/Missing/Do not know); Residence (urban and rural); region (north, central, and south); wealth (5 quintiles); number of children ever born (4 categories of none, 1–2, 3–4, and 5–12); media exposure (2 categories of yes/no computed based on the weekly frequency of reading newspaper/magazine, listening to radio, and watching TV. Respondents reporting as “not at all” or those who did not respond to all three questions were categorized as having no exposure); and frequency of alcohol use (2 categories of yes/no, in terms of having taken one or more drinks on any number of days in the past month as affirmative, while no alcoholic drink on any day or non-response were categorized as not having taken any drink). For women, one additional variable of age difference between her and her husband was also examined and coded in four categories of either husband/partner being younger or of the same age, 1–4 years, 5–9 years, or 10 or more years older. Two women answered as “Do not know” to the question about their husband/partner age and these two cases were assigned the median age of the sample to ensure that no record would be dropped in the sample and multivariable logistic regression models. All these correlates were chosen *a priori* based on prior evidence (5–28) and availability in the LSIS III.

### Statistical analysis

The de-identified female and male data files from the LSIS III were downloaded in SPSS format and converted to Stata for secondary analysis using Stata version 18 (Texas, United States). Analysis accounted for the complex survey design; survey design was declared using “PSU_SE” and “strata,” and the weight variable “mnweight” for men and “wmweight” for women, respectively for the primary sampling unit, strata, and weights. A *p* < 0.05 was deemed statistically significant. Analysis was restricted to those individuals who were either currently married or living with an intimate partner.

Data files for males and females were analyzed separately in a three-step analysis entailing descriptive statistics, binary simple logistic regression models, and multivariable logistic regression models. Specifically, this three-step approach was used to first describe the sample and provide context, then to run bivariable models to screen candidate correlates, and finally to estimate independent associations in the final multivariable model. The simple binary logistic regression models were used to compute the association between the outcome variable of WBA and each of the explanatory variables individually. Those variables that were found to be associated with WBA at the statistical significance level of ≤ 0.20 were included in the multivariable logistic regression model. However, variables could also change in statistical significance after adjustment due to confounding or suppression effects in the multivariable model. Results of these models were reported as crude and adjusted odds ratios, 95% confidence intervals, and their statistical significance. For the final multivariable model for women and men, statistical significance was determined at the level of <0.05. Model goodness-of-fit (Archer-Lemeshow test) and Variance Inflation Factors (VIF) were computed separately for both women and men; VIF of less than 5 was used as the cutoff value.

## Results

A total of 16,596 women and 6,868 men reported being either currently married or cohabiting in the LSIS III. Eleven correlates of WBA for women and 10 correlates for men were examined. Tables 1, 2, respectively, provide descriptive statistics for female and male respondents in terms of WBAs and its correlates. Among currently married or partnered women aged 15–49 in Lao PDR, 13.5% (95% CI: 12.6–14.5) agreed that a husband is justified in beating his wife under at least one of five specified circumstances. The most commonly accepted justification was “if she argues with him” (10.0%), followed by “if she neglects the children” (8.5%), “if she goes out without telling him” (6.3%), “if she refuses sex with him” (4.9%), and “if she burns the food” (2.1%). Among men, 11.0% (95% CI: 9.5–12.8) endorsed at least one justification for wife beating, with the highest endorsement also being for “if she argues with him” (8.1%).

**Table 1:** Counts and proportions of study variables in currently married/partnered women—Lao MICS 2023.

Variable	Unweighted count (*N* = 16,596)	Percentage (weighted)
Outcome variable		
Husband justified in beating wife		
(Any one or more of five)	Yes = 2,385	13.51 (12.57–14.51)
*If she goes out without telling him*	*Yes = 979*	*6.29 (5.58–7.09)*
*If she neglects the children*	*Yes = 1,397*	*8.52 (7.72–9.39)*
*If she argues with him*	*Yes = 1,783*	*9.99 (9.25–10.78)*
*If she refuses sex with him*	*Yes = 864*	*4.88 (4.39–5.43)*
*If she burns the food*	*Yes = 349*	*2.10 (1.80–2.45)*
Explanatory variables		
Age	15–19y = 1,100	6.05%
	20–24y = 2,273	12.72%
	25–29y = 2,909	16.72%
	30–34y = 3,163	18.73%
	35–39y = 3,040	18.63%
	40–44y = 2,458	15.98%
	45–49y = 1,653	11.18%
Respondent’s education	None/pre-primary = 2,809	16.50%
	Primary = 5,784	34.61%
	Secondary = 5,966	35.88%
	Post-secondary/higher = 2,037	13.01%
Ethnicity of household head	Lao-Tai = 8,558	61.58%
	Mon-Khymer = 4,817	24.97%
	Hmong-Mein = 2.139	9.51%
	Chinese-Tibetan/missing/DK = 1,082	3.94%
Residence	Urban = 4,917	31.04%
	Rural = 11,679	68.96%
Region	North = 6,601	32.56%
	Central = 6,513	48.97%
	South = 3,482	18.47%
Wealth	Poorest = 3,817	19.80%
	Second/poorer = 3,873	20.33%
	Middle = 3,279	20.15%
	Fourth/richer = 3,000	20.26%
	Richest = 2,627	19.46%
Husband/partner has more wives/partners	Yes = 243	1.33%
	No/no-response = 16,353	98.67%
Children	0 = 1,568	9.90%
	1–2 = 8,753	54.37%
	3–4 = 4,823	27.58%
	5–12 = 1,452	8.14%
Media exposure	No = 6,871	36.92%
	Yes = 9,725	63.08%
Age difference	Younger or same as wife/partner = 3,814	22.48%
	1–4 years = 6,965	42.48%
	5–9 years = 4,167	25.00%
	10 or more years = 1,650	10.05%
Alcohol use in past month		
	Zero days = 9,063	54.51%
	1–30 days = 7,533	45.49%

**Table 2:** Counts and proportions of study variables in currently married/partnered men—Lao MICS 2023.

Variable	Unweighted count (*N* = 6,868)	Percentage (weighted)
Outcome variable		
Husband justified in beating wife		
(Any one or more of five)	Yes = 916	11.02 (9.50–12.75)
*If she goes out without telling him*	*Yes = 437*	*3.75 (3.20–4.38)*
*If she neglects the children*	*Yes = 493*	*4.92 (4.07–5.94)*
*If she argues with him*	*Yes = 624*	*8.13 (6.74–9.77)*
*If she refuses sex with him*	*Yes = 277*	*2.53 (2.01–3.17)*
*If she burns the food*	*Yes = 170*	*1.74 (1.34–2.26)*
Explanatory variables		
Age	15–19y = 180	2.53%
	20–24y = 735	9.78%
	25–29y = 1,085	14.82%
	30–34y = 1,342	19.11%
	35–39y = 1,417	21.22%
	40–44y = 1,207	18.47%
	45–49y = 902	14.08%
Respondent’s education	None/pre-primary = 505	7.09%
	Primary = 2,191	32.01%
	Secondary = 2,895	42.40%
	Post-secondary/higher = 1,277	18.50%
Ethnicity of household head	Lao-Tai = 3,395	59.15%
	Mon-Khymer = 2,065	26.07%
	Hmong-Mein = 941	10.57%
	Chinese-Tibetan/missing/DK = 467	4.22%
Residence	Urban = 1,898	29.34%
	Rural = 4,970	70.66%
Region	North = 2,740	33.05%
	Central = 2,619	48.34%
	South = 1,509	18.60%
Wealth	Poorest = 1,676	21.49%
	Second/poorer = 1,665	21.28%
	Middle = 1,334	19.68%
	Fourth/richer = 1,164	19.33%
	Richest = 1,029	18.22%
Other wives or live-in partners		
	Yes = 1,277	20.37%
	No/No response = 5,591	79.63%
Children	0 = 707	11.29%
	1–2 = 3,643	54.83%
	3–4 = 1,913	25.94%
	5–12 = 605	7.94%
Media exposure	No = 2,365	31.49%
	Yes = 4,503	68.51%
Alcohol use in past month		
	Zero days = 1,646	23.30%
	1–30 days = 5,222	76.70%

Regarding sociodemographic characteristics, over half of women were aged 15–34 years (54.22%), while men in this age range comprised 46.24% of the male sample. Most women (70.49%) and men (74.41%) had attained either primary or secondary education. Lai-Tai ethnicity of the household head was more frequent for both women (61.58%) and men (59.15%). Over two-thirds of women (68.96%) and men (70.66%) lived in rural areas. A little under half (48.97%) of women and men (48.34%) belonged to the Central region of the country. The poorest, second, and middle wealth quintile, i.e., the lowest three quintiles were reported by 60.28% of women and 62.45% of men. Zero lifetime births were reported by 9.90% women, while 11.29% men reported not having any children.

Media exposure was, respectively, reported by 63.08 and 68.51% of women and men. Alcohol use was reported by 45.49% of women and 76.70% of men. Notably, 20.37% of men reported having more than one wife or live-in partner, while only 1.33% of women reported that their husband or partner has more than one wife or partner. Regarding age difference between wife/partner, 35.05% women reported that their husband/partner was five or more years older than them.

Tables 3, 4, respectively, provide results of the simple and multivariable logistic regression models for female and male respondents in terms of WBAs and its correlates. Simple and multivariable logistic regression model results in terms of crude and adjusted odds ratios and their statistical significance, along with their corresponding 95% confidence intervals, are, respectively, reported in Tables 3, 4 for women and men. For men, only urban/rural residency status was not found to be statistically significantly associated with the WBA outcome variable at the *p* ≤ 0.20. Hence, 10 covariates were included in the final multivariable logistic regression model for women and nine for men.

**Table 3:** Crude and adjusted odds ratios for associations between wife-beating attitudes and selected variables in currently married/partnered women—Lao MICS 2023.

Explanatory variable	Unadjusted OR	*p*-value	95% CI	Adjusted OR	*p*-value	95% CI
Age						
15–19	Reference			Reference		
20–24	0.85	0.142	0.68–1.06	0.96	0.740	0.77–1.21
25–29	0.84	0.149	0.67–1.06	1.13	0.333	0.89–1.43
30–34	0.76	0.013	0.60–0.94	1.05	0.687	0.81–1.37
35–39	0.73	0.006	0.59–0.91	1.05	0.736	0.81–1.36
40–44	0.66	0.001	0.53–0.84	0.93	0.628	0.71–1.23
45–49	0.65	0.001	0.50–0.84	0.93	0.607	0.69–1.24
Respondent’s education						
None/pre-primary	Reference			Reference		
Primary	0.93	0.524	0.75–1.16	1.24	0.048	1.002–1.54
Secondary	0.87	0.192	0.71–1.07	1.19	0.148	0.94–1.49
Post-secondary/higher	0.45	<0.001	0.34–0.61	0.79	0.128	0.58–1.07
Ethnicity of household head						
Lao-Tai	Reference			Reference		
Mon-Khymer	1.61	<0.001	1.32–1.97	1.40	0.004	1.12–1.76
Hmong-Mein	2.26	<0.001	1.81–2.84	1.54	0.002	1.18–2.02
Chinese-Tibetan/						
Missing/DK	1.33	0.261	0.81–2.20	1.11	0.674	0.68–1.81
Residence						
Urban	Reference			Reference		
Rural	1.43	<0.001	1.18–1.72	1.10	0.392	0.89–1.36
Region						
North	Reference			Reference		
Central	1.06	0.548	0.88–1.26	1.34	0.002	1.11–1.61
South	0.38	<0.001	0.29–0.48	0.42	<0.001	0.32–0.54
Wealth						
Poorest	Reference			Reference		
Second/Poorer	0.71	<0.001	0.59–0.86	0.82	0.035	0.69–0.99
Middle	0.64	<0.001	0.51–0.80	0.83	0.116	0.65–1.05
Fourth/richer	0.60	<0.001	0.48–0.75	0.83	0.145	0.65–1.06
Richest	0.38	<0.001	0.29–0.49	0.57	<0.001	0.42–0.76
Husband/partner has more wives/partners						
Yes	Reference			Reference		
No	0.76	0.173	0.51–1.13	0.72	0.112	0.48–1.08
Children						
No children	Reference			Reference		
1–2 children	0.98	0.862	0.81–1.19	1.02	0.832	0.83–1.25
3–4 children	1.004	0.971	0.82–1.23	0.94	0.567	0.74–1.18
5–11 children	1.33	0.021	1.04–1.69	0.97	0.856	0.73–1.30
Media exposure						
No	Reference			Reference		
Yes	0.66	<0.001	0.58–0.77	0.83	0.015	0.71–0.96
Age difference						
Younger or same as wife/partner	Reference			Reference		
1–4 years	0.90	0.127	0.79–1.03	0.89	0.069	0.78–1.01
5–9 years	0.89	0.156	0.76–1.04	0.87	0.095	0.75–1.02
10 or more years	0.80	0.031	0.65–0.98	0.78	0.018	0.63–0.96
Alcohol use in past month						
Zero days	Reference			Reference		
1–30 Days	0.82	0.007	0.71–0.95	0.95	0.477	0.82–1.10

OR, odds ratio.

**Table 4:** Crude and adjusted odds ratios for associations between wife-beating attitudes and selected variables in currently married/partnered men—Lao MICS 2023.

Explanatory variable	Unadjusted OR	*p*-value	95% CI	Adjusted OR	*p*-value	95% CI
Age						
15–19	Reference			Reference		
20–24	0.43	0.001	0.26–0.70	0.40	<0.001	0.24–0.66
25–29	0.41	0.001	0.24–0.68	0.40	0.001	0.24–0.68
30–34	0.36	<0.001	0.21–0.63	0.39	0.001	0.22–0.69
35–39	0.38	<0.001	0.23–0.63	0.41	0.002	0.23–0.72
40–44	0.36	<0.001	0.21–0.62	0.39	0.002	0.22–0.71
45–49	0.39	0.002	0.21–0.70	0.42	0.009	0.22–0.81
Respondent’s education						
None/pre-primary	Reference			Reference		
Primary	0.73	0.193	0.45–1.17	1.07	0.760	0.68–1.70
Secondary	0.69	0.170	0.41–1.17	1.24	0.396	0.76–2.02
Post-secondary/higher	0.57	0.049	0.32–0.998	1.57	0.088	0.93–2.65
Ethnicity of household head						
Lao-Tai	Reference			Reference		
Mon-Khymer	1.98	0.001	1.31–2.97	1.52	0.053	0.99–2.31
Hmong-Mein	2.20	<0.001	1.43–3.38	1.42	0.150	0.88–2.27
Chinese-Tibetan/Missing/DK	1.84	0.003	1.23–2.76	1.48	0.128	0.89–2.44
Residence						
Urban	Reference			Not Applicable		
Rural	1.15	0.493	0.78–1.69			
Region						
North	Reference			Reference		
Central	1.25	0.163	0.91–1.72	1.36	0.097	0.95–1.96
South	0.63	0.010	0.44–0.90	0.66	0.045	0.44–0.99
Wealth						
Poorest	Reference			Reference		
Second/Poorer	0.89	0.487	0.65–1.23	1.06	0.700	0.80–1.39
Middle	0.65	0.035	0.43–0.97	0.83	0.294	0.58–1.18
Fourth/richer	0.49	0.002	0.32–0.76	0.70	0.101	0.46–1.07
Richest	0.35	<0.001	0.22–0.55	0.48	0.003	0.30–0.78
Other wives or live-in partners						
Yes	Reference			Reference		
No	2.35	<0.001	1.66–3.2	2.24	<0.001	1.57–3.20
Children						
No children	Reference			Reference		
1–2 children	0.93	0.656	0.67–1.29	1.25	0.196	0.89–1.77
3–4 children	1.04	0.825	0.74–1.46	1.23	0.290	0.84–1.78
5–11 children	2.25	<0.001	1.53–3.33	2.25	<0.001	1.44–3.51
Media exposure						
No	Reference			Reference		
Yes	0.54	<0.001	0.42–0.71	0.70	0.002	0.56–0.87
Alcohol use in past month						
Zero days	Reference			Reference		
1–30 days	1.35	0.046	1.005–1.83	1.52	0.002	1.17–1.98

OR, odds ratio.

Both multivariable models demonstrated good fit, with non-significant results: women {[*F*(9, 965)] = 0.31; *p*-value: 0.9724}, and men {[F(9, 965)] = 0.99; *p*-value: 0.4475}. Variance inflation factors were below 2.57 in both models, indicating no concerning multicollinearity.

Among women, educational attainment, the household head’s ethnicity, region of residence, household wealth, media exposure, and the age difference with their husband/partner were significantly associated with WBA in the multivariable model. Compared to women with no or pre-primary education, women with a primary education had higher odds of accepting wife beating (aOR: 1.24; 95% CI: 1.002–1.54). Compared to Lao-Tai ethnicity of the household head, women with Mon-Khymer (aOR: 1.40; 95% CI: 1.12–1.76) or Hmong-Mein (aOR: 1.54; 95% CI: 1.18–2.02) household heads had higher odds of accepting wife beating. Compared to residency in the north region, women residing in the central region had higher odds of WBA endorsement (aOR: 1.34; 95% CI: 1.11–1.61), whereas those in the south had significantly lower odds of endorsing WBA (aOR: 0.42; 95% CI: 0.32–0.54). Compared with the lowest/poorest quintile of wealth, women in the second/poorer quintile had lower odds of WBA acceptance (aOR:0.82; 95%CI: 0.69–0.99), as well as women in the highest/richest quintile (aOR:0.57; 95%CI: 0.42–0.76). Compared with women whose husband/partner were of the same age or younger, those women whose husband/partner were 10 or more years older had reduced acceptance of WBA (aOR:0.78; 95%CI: 0.63–0.96). Finally, compared with women with no media exposure, those with some exposure were associated with reduced acceptance of WBA (aOR:0.83; 95%CI: 0.71–0.96).

For men, the correlates of age, region of residence, household wealth, having other wives and live-in partners, number of children ever born, media exposure, and alcohol use were statistically significantly associated with WBA in the multivariable model. Compared to men aged 15–19, all older age groups had significantly lower odds of WBA acceptance, with adjusted odds ratios ranging from 0.39 to 0.42. Compared to residency in the north region, men residing in the south region had lower odds of WBA endorsement (aOR: 0.66; 95% CI: 0.44–0.99). Men in the richest quintile were significantly less likely to justify wife beating (aOR: 0.48; 95% CI: 0.30–0.78), compared with the poorest quintile. Interestingly, men who reported not having other wives or live-in partners had over twice the odds of endorsing wife beating (aOR: 2.24; 95% CI: 1.57–3.20), compared with those who did. Media exposure was again protective (aOR: 0.70; 95% CI: 0.56–0.87). Having 5–11 children (aOR: 2.25; 95% CI: 1.44–3.51), compared with no having children, and alcohol use in the past month (aOR: 1.52; 95% CI: 1.17–1.98) were associated with increased odds of WBA acceptance.

## Discussion

This study addressed the evidence gap in Laos regarding WBA by utilizing the most recent MICS survey data. This is the first study reporting multivariable analyses of WBA acceptance among women and men using the most recent nationally and sub-nationally representative data from Lao PDR. However, WBA acceptance does not necessarily construe actual IPV experiences of women or actual perpetration of IPV by men who harbor such attitudes. The results of 13.51% of women and 11.02% of men currently married/partnered who accept WBA are concerning. Over the course of three LSIS from 2011 to 2023 in Lao DPR, remarkable progress has been made in terms of substantially reducing the burden of WBA acceptance in both women and men. Nonetheless, their prevalence is still in double digits in both men and women and, as a women’s human rights issue, it is never acceptable to physically beat wives under any circumstances. Attitudes are acquired—not inborn—and, as such, are amenable to modification and reversal through guided, targeted, and sustained health education and promotion endeavors. And results of the multivariable models suggest correlates of WBA acceptance that may help identify targeted population segments.

The higher proportion of WBA acceptance in women compared with men is a consistently reported finding (21, 22, 28). However, mixed results have also been reported (16). Effective addressal of the public health challenge of IPV needs to include changing such entrenched attitudes. For both women and men, the prevalence of the five WBA acceptance reasons was ranked the same: that is, the most common WBA was if she argues with him, followed by neglecting the children, goes out without telling her husband, refusing sex with him, and burning food. Although some of the confidence intervals for WBAs overlapped within both women and men, this nonetheless suggests fairly uniform, socially ingrained norms regarding WBAs. The leading WBA in both sexes—arguing with the husband—suggests a social norm that dictates submissiveness to the husband. The second most common WBA—neglecting children—and the two least commonly held attitudes—refusing sex and burning food—point toward traditional expectations for women to live their lives as wives. The third most common WBA—acceptance of a wife going out without telling her husband—suggests a sense of entitlement among husbands to control their wives’ lives. Collectively, acceptance of these WBAs suggests old practices rooted in socially harmful norms granting husbands the right to control the lives of their wives.

Several studies using DHS data have reported an association between WBA acceptance and IPV (5–7). However, the cross-sectional study design of these surveys does not imply causality—merely an association that could be bidirectional or a case of reverse causation. Nonetheless, this association is concerning and underscores the importance of addressing WBA acceptance as part of IPV control and prevention efforts. This association was also echoed in the only nationally representative survey of IPV conducted in 2014 in Laos, which reported an association between WBA acceptance and IPV, albeit using different questions on attitudes from those used in DHS and MICS (26).

Numerous studies using MICS and DHS data have reported an association of higher household wealth with lower acceptance of WBA in women (13), and, where studied, in both genders (16, 20, 22), albeit one study based on 20 countries reported mixed results as well (17). This perhaps reflects more exposure to and acceptance of progressive norms in wealthier families. In this study, compared with the poorest quintile, women in the second (poorer) and fifth (richest) quintiles had significantly lower odds of accepting WBA, indicating a protective effect of higher household wealth on rejecting such attitudes. For men, wealth was not statistically significantly associated with WBA. A lack of such association in men may reflect gender-specific pathways through which wealth may not influence WBA attitudes. Perhaps men’s WBA acceptance may be much more strongly shaped by deeply entrenched gender norms.

Similarly, several studies have reported an association between higher educational attainment and lower acceptance of WBAs among women (10–15), men (17, 23), and both genders (20, 22). One study using DHS data for Ghana studied both genders but found this association in women only (21). Higher educational attainment might lead to more acceptance of gender equity values. Results from Lao MICS 2023 show that educational attainment in men was not found to be significantly associated with WBA acceptance. While in women it was only significant for those who had a primary education, compared with those who had no or pre-primary education only. This is possibly due to attenuation of educational attainment by other covariates in the multivariable model for men.

Other findings of multivariable models for women and men were also rather contradictory. For women, age was not statistically significantly associated with the acceptance of WBA, while in men, compared with the youngest age group of 15–19 years, all other six age-groups—spanning 5-year intervals—were statistically significantly associated with lower acceptance of WBA. For women, the crude/unadjusted odds ratios of association with WBA acceptance were lower, but statistical significance was only found for those grouped in 30–49 years. For the two age groups spanning 20–29 years, crude odds ratios were lower for WBA acceptance but did not reach statistical significance. This suggests that women and men may be socialized differently with age regarding gender norms. Secondly, in women, age effect is attenuated by the influence of other covariates in the multivariable model. Several studies report an association of older age with lower acceptance of WBA in both women and men (13, 15, 16, 20, 23). One study reported this association in women but not men (21), and another study reported an association of older age with higher acceptance of WBA in women (12).

Ethnicity was statistically significant for women only and compared with women in households headed by Lao-Tai, those in Mon-Khymer and Hmong-Mein were significantly more likely to accept WBA. Ethnic groups may differ in cultural norms on WBA and perhaps women’s attitudes may be more influenced by their ethnicity than men’s. Perhaps, a household’s ethnicity may reflect power dynamics that only influence and shape women’s attitudes. Ethnicity has been found to be a statistically significant correlate of WBAs in several studies (10–13).

Residence, in terms of urban/rural location and north, central, and south regions, was inquired about in the LSIS III. For women, urban/rural residence was not statistically significantly associated with WBA, while for men it even failed the inclusion criteria threshold in the multivariable model. Compared to the north region, women in the central region had higher odds of accepting WBA, while those in the south had lower odds; for men, region was not statistically significant. The importance of region as a geographical administrative subdivision of a country has been reported to be associated with WBA (12, 15, 20, 21). Geographic variation in WBAs may reflect a mix of structural (education, economy, and institutions), cultural (traditions, religion, and ethnicity), and environmental (urbanization and media exposure) influences. The association of rural residency with higher WBA acceptance has been a consistent finding (12, 16, 17, 20, 22). In this study, the lack of such association might be due to regional heterogeneity or its effect being mediated by other covariates so its independent contribution diminishes or is lost.

Women’s acceptance of WBA was not significantly associated with whether their partners had other wives or partners. In contrast, compared with men who had more than one wife or partner, those with only one wife or partner had over twice the odds of accepting WBA. This may suggest that men in monogamous relationships are not less likely to hold traditional patriarchal views. However, this finding warrants further investigation. In women, internalization of norms may occur regardless of relationship structure, resulting in reduced detectable differences. However, a study based on 10 East African countries using DHS data reported that men with more wives had higher odds of WBA acceptance compared to those who had one wife (23). While another study based on 21 Sub-Saharan African countries in women reported that women whose husbands/partners had other wives had higher odds of accepting WBA (18).

The association of WBA acceptance with the number of children for women was not statistically significant. However, compared to men with no children, those who had 5–11 children ever born had twice the odds of accepting WBA attitudes. This might be explained by different role expectations, as childbearing may not alter women’s attitudes toward WBA, but a larger family size might be tied to traditional roles and acceptance of dominance and control by men. However, the role of number of children and its association with WBA is equivocal. One study reported that women who had given birth are less accepting of WBA (10), while another study reported that having more children leads to higher WBA acceptance in both sexes (20); no association in men has also been reported (23).

Media exposure’s association with lower acceptance of WBA has been reported in both women and men (10, 11, 21, 22). This study concurs with that finding in both sexes using MICS data from Laos. However, a lack of this association in both sexes has also been documented (13, 20, 23). Media exposure raises awareness and challenges traditional norms, and thus might act as an empowerment pathway. However, in the presence of other covariates in the multivariable model, it may lose its discerning ability, in addition to heterogeneity of media content and cultural resistance to deeply ingrained norms.

Men who used alcohol for one or more days in the past 30 days had higher odds of accepting WBA compared with those who did not, while in women alcohol use failed to reach the level of statistical significance. Alcohol use in men may increase aggressive tendencies and acceptance of violence, while drinking contexts may expose men to peers who normalize WBA. In contrast, female drinking might be stigmatized, thus limiting both its prevalence and its influence on WBA. Finally, regarding the age difference between a woman and her partner and its association with WBA, compared to women whose husbands/partners were the same age or younger, those whose husbands/partners were 10 or more years older than them had lower odds of accepting WBA. One possible explanation is that perhaps older husbands/partners are more protective or less accepting of violence toward their wives. Or perhaps large age gaps may reduce direct conflict, lowering justification for violence. One study looked at the age difference between married couples and reported that women 6–10 years younger than their husbands were less accepting of WBAs versus those who were of the same age or had less than 6 years’ age difference with their husbands. However, the WBAs of women who were 11 or more years younger than their husbands were not found to be statistically significant (10). The association of a larger age difference with the male partner showed a protective effect, with lower odds of WBA acceptance in women. This may reflect different relationship dynamics, e.g., differences in decision-making roles. However, the cross-sectional nature of the survey does not allow any causal inferences, only associations. Future studies using a longitudinal design with more nuanced survey instruments would be better suited to address causality and underlying mechanisms.

These findings should be interpreted with caution, as empirical evidence regarding the underlying mechanisms—particularly in the Laos context—remains limited. The proposed explanations are speculative and may guide future research. WBAs are a marker of social acceptance of physical IPV and reflect gender inequality. Men’s acceptance may reflect higher normative support for IPV perpetration and control, while women’s acceptance may reflect internalized gender norms with a resultant lower ability to question such attitudes. The reported gender differences in WBA correlates differed by gender: among women, WBA acceptance was associated with education, ethnicity of the household head, region, wealth, media exposure, and the age difference with husband/partner; among men it was associated with age, region, wealth, having other wives/live-in partners, number of children ever born, media exposure, and alcohol use.

It is important to underscore that WBA prevalence does not imply or equate IPV prevalence, as the Lao PDR MICS did not inquire about IPV. Secondly, IPV entails emotional and sexual violence in addition to physical violence. However, wife beating is one manifestation of physical IPV, which also includes pushing, pulling hair, burning, etc.

The main strength of the study is the use of the most recent nationally and subnational representative data on WBA in the Lao PDR for the prevalence and correlates using separate multivariable models for both women and men. The limitations include computing associations of WBA with various factors (i.e., correlates), as the cross-sectional study design does not lend itself to determining causality. Social desirability bias, even when using CAPI, does not rule out this bias in responses. Other limitations include a sample restricted to those aged 15–49, and to currently-married/partnered respondents. Hence, individuals older than 49 years and those not currently married/partnered were not included by design; therefore, the estimated WBA prevalence may not be representative of the entire adult population. Finally, assessment of cultural mechanisms underpinning WBA attitudes would necessitate qualitative and mixed-methods research.

Future iterations of MICS in Laos need to include questions on attitudes pertaining to all three types of IPV—i.e., emotional, physical, and sexual—as well as on actual IPV experiences among both men and women.

Differing regional and ethnic variations in WBA acceptance among women and men augur the need for a more nuanced understanding and efforts to reverse such acceptance. Empowering women and men through greater and better media exposure, including scaling up media-based prevention messaging on alcohol-related harm reduction, can increase their awareness of women’s rights and recourse to support and foster a better appreciation and more enlightened thinking about what constitutes acceptable behavior in marriage.

## Conclusion

The double-digit prevalence of WBA acceptance among both men and women, as shown in the 2023 LSIS III data from Lao PDR, remains a serious public health concern. Media exposure in both women and men was associated with reduced acceptance of WBA. This suggests that targeted media-based health promotion could be a feasible intervention entry point. Regional and ethnic variation in wife-beating acceptance indicates the need for prioritized and culturally appropriate interventions tailored to higher-burden subgroups.

## Data Availability

Publicly available datasets were analyzed in this study. This data can be found at: The United Nations Children’s Fund (UNICEF) via its Multiple Indicator Cluster Survey (MICS) website (https://mics.unicef.org/) granted approval for the secondary analysis reported in this study to the author and provided deidentified data files for the Lao Social Indicator Survey III (LSIS III), 2023. Author did not have access to information that could identify individual survey participants.
